# Supplementary material for: Chronic stress causes striatal disinhibition mediated by SOM-interneurons in male mice
Source: Nat Commun. 2022 Nov 29;13:7355. doi: 10.1038/s41467-022-35028-4 (PMC9709160; doi:10.1038/s41467-022-35028-4)
Supplement: Supplementary file 1 — Supplementary Information [file 41467_2022_35028_MOESM1_ESM.pdf]

Supplementary Information

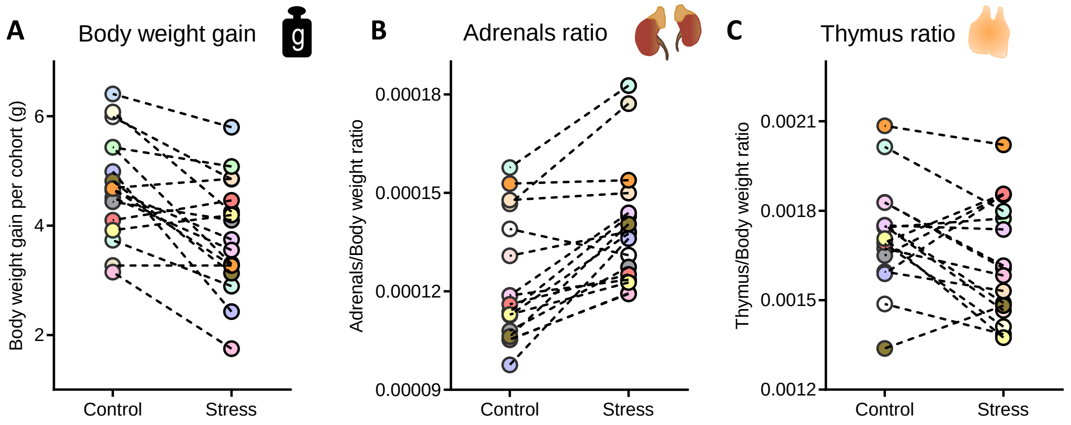

Sup. Fig.1 - Physiological parameters grouped *per* cohort.

(A) Mean value of body weight gain for each experimental cohort of stress and control mice. Each colored circle represents an experimental cohort in which littermate mice were randomly assigned to the control or CS group.
(B) Mean value per cohort of adrenal glands weight normalized to body weight.
(C) Mean value per cohort of thymus glands weight normalized to body weight.
Each color represents the same cohort in panels A, B and C. n=17 control and n=17 stress cohorts.

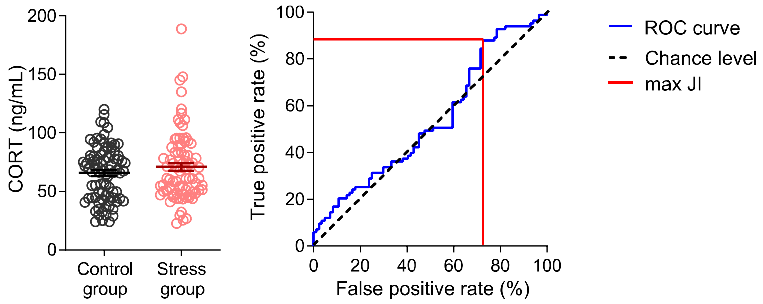

Sup. Fig.2 - Circulating level of corticosterone after 21 days of CS protocol.

(Left panel) Circulating blood level of corticosterone determined by ELISA for control (black) and stress (red) mouse after 21 days of CS protocol. Data are mean ± SEM; n=84 control and n=83 chronic stress mice; Welch's unpaired t-test; p-value 0.1997.

(Right Panel) Receiver operating characteristic (ROC) curves (blue line) for corticosterone levels data and maximum Youden J index (JI; red line). Dashed line represents chance level. Data reveal poor predicting power for corticosterone with its ROC curve closely matching chance level curve.

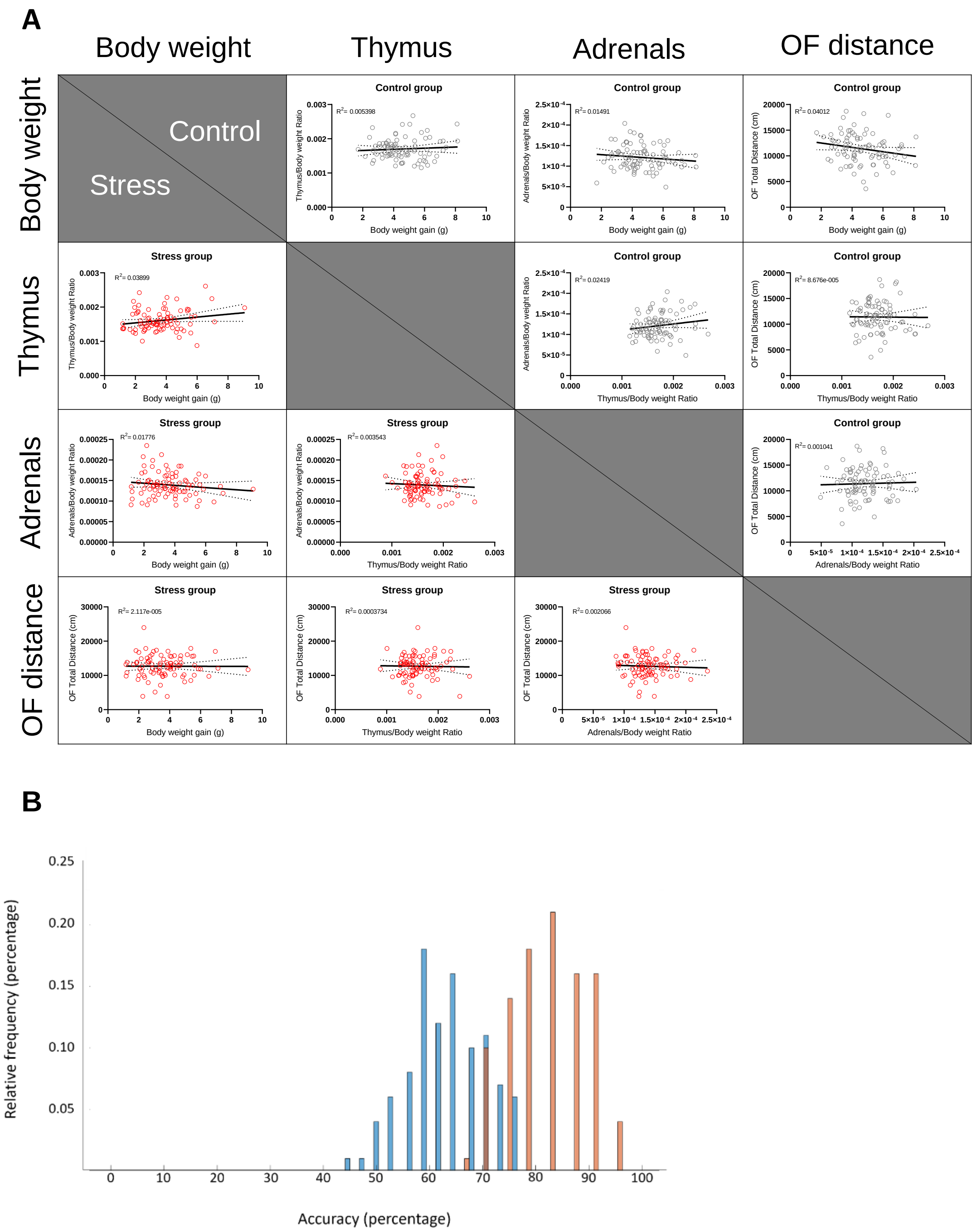

Sup. Fig.3 – Correlation matrix of stress protocol outcome measures.

A) Scatterplots of all pair-wise correlations between the stress protocol outcome (body weight gain, thymus weight and adrenals weight) and behavior (OF distance travelled) measures, for the control and stress group. Fitted linear regression (black line) with 95% confidence intervals (dashed lines) to all scatterplots shows no significant correlation for any possible pair of outcome measures (Control group (gray): thymus vs body R^2=0.005; adrenals vs body R^2=0.015; OF vs body R^2=0.04; adrenals vs thymus R^2=0.02; OF vs thymus R^2=8.7x10^{-5}; OF vs adrenals R^2=0.001; Stress group (red): thymus vs body R^2=0.04; adrenals vs body R^2=0.02; adrenals vs thymus R^2=0.004; OF vs body R^2=2.1x10^{-5}; OF vs thymus R^2=0.0004; OF vs adrenals R^2=0.002). B) Histogram of SVM classifier accuracy to classify mice as controls or stressed, using all physiological measures before (blue) and after D-scoring (orange) for 100 repetitions (randomly assigned training and test sets; holding out 20% of data for testing). Mean accuracy improves from 63% before D-scoring to 82% after D-scoring.

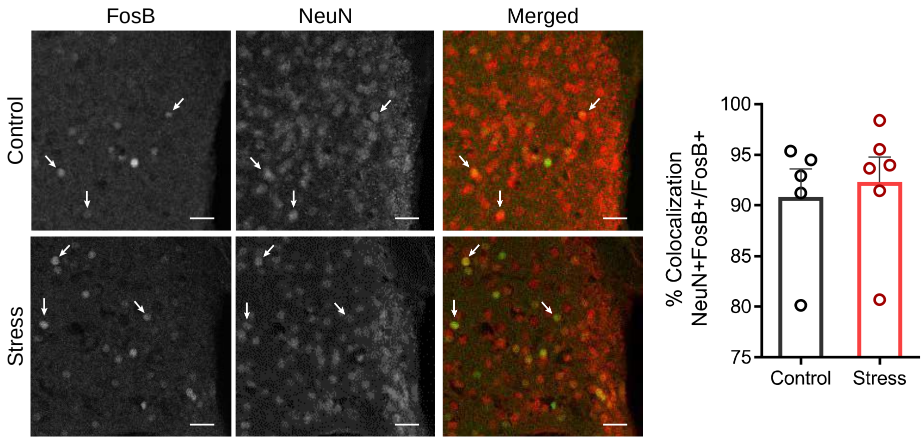

Sup. Fig.4 - FosB/∆FosB and NeuN co-staining in DMS of control and stress mice.

Representative immunohistochemistry images of FosB/ΔFosB (green) and NeuN (red) co-staining in dorsomedial striatum (DMS) of control and stressed mice. Percentage of double positive NeuN+FosB/ΔFosB cells *per* FosB/ΔFosB positive cells in control and stressed mice reveals approx. 90% colocalization (each dot represents one mouse; both hemispheres were quantified in 4 brain slices per mouse). Data are mean ± SEM; n=5 control and n=6 chronic stress mice; Welch's unpaired t-test. Scale bar=25 µm.

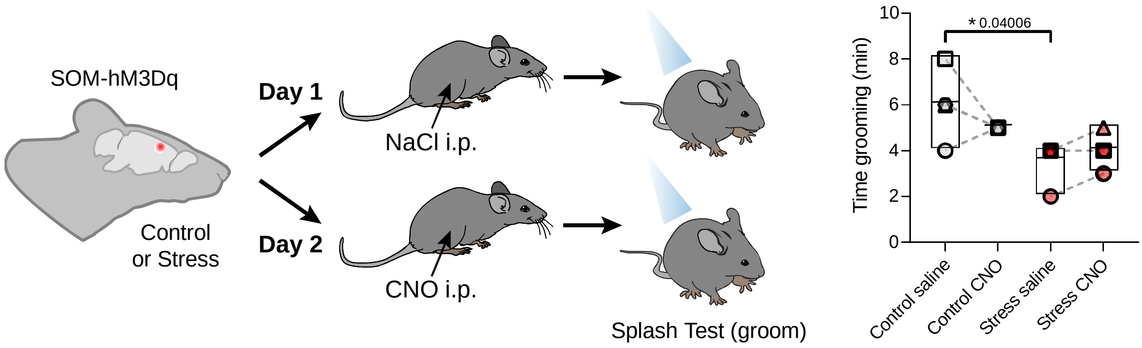

Sup. Fig.5 – Chronic stress mice display reduced grooming in splash test.

The effect of stress and striatal SOM activation on grooming behavior was assessed by performing the splash test on control and stressed mice. Left) Adenovirus expressing DIO-hM3D(Gq)-mCherry was bilaterally injected in dorsomedial striatum region (DMS) of *SOM-Cre* mice that were randomly assigned to the control or stress group. After 21 days of CS protocol, mice from both groups received i.p. injection of saline on Day 1 and CNO on Day 2, and performed the splash text approx. 1 h after the injection. Right) Summary bar graph reveals significantly reduced grooming time in stressed mice on Day 1 when compared with controls (unpaired t-test; *p= 0.04, n=4 control and n= 4 stress mice). After CNO injection on Day 2, no significant differences were found between saline and CNO for both groups (paired t-test). Despite not reaching statistical significance, a trend for increased grooming time can be observed in the stressed group upon striatal SOM activation, whereas no such trend was observed in the control group. Data are represented by floating min/max boxes with overlaid individual data points and line at the mean value. Credit: image was adapted from https://scidraw.io.

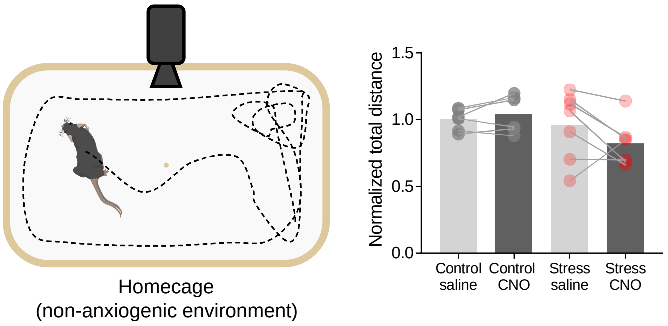

Sup. Fig.6 – Chronic stress mice do not display hyperlocomotion in homecage environment.

The effect of striatal SOM activation on locomotion was assessed by measuring total distance travelled in a homecage. Left) Adenovirus expressing DIO-hM3D(Gq)-mCherry was bilaterally injected in dorsomedial striatum region (DMS) of *SOM-Cre* mice that were randomly assigned to the control or stress group. After 21 days of CS protocol, mice from both groups received i.p. injection of saline on Day 1 and CNO on Day 2, and were videotaped in homecage approx. 30min after the injection. Right) Summary bar graph reveals no differences in total distance travelled between control and stress mice, nor between Day 1 and Day 2. Data are mean ± SEM; n=6 control and n=7 chronic stress mice. Despite not reaching statistical significance, a trend for decreased locomotion can be observed in the stressed group upon striatal SOM activation, whereas no such trend was observed in the control group. Credit: image was adapted from https://scidraw.io.

Supplementary Methods

FosB/ΔFosB and NeuN immunohistochemistry:
Mice were transcardially perfused with saline followed by 4% paraformaldehyde (PFA). Brains were extracted, post-fixed by overnight immersion in 4% PFA and then transferred to 30% sucrose in phosphate-buffered saline (PBS) solution for 24 h (immersion at 4°C). Brains were then embedded in optimal cutting temperature compound (OCT; Bio-Optica) and 30 µm-thick coronal sections of the striatum were serially cut in the cryostat (Leica Microsystems). Sections were washed three times for 10 min with PBS and placed in citrate buffer at 80°C for 20 min. After that, brain sections were allowed to cool down at room temperature (RT) for 20 min and then washed 3 times for 10 min with PBS. Brain sections were permeabilized twice with 0.3% Triton X-100 (Sigma–Aldrich) in PBS for 10 min at RT. After washing 3 times in PBS for 10 min, brain sections were blocked using 15% NGS, 5% BSA, 0.2% Triton-X (blocking buffer) for 1h at RT. Blocked sections were incubated overnight with primary antibody for FosB (Rabbit, #2251, Cell Signaling, 1:200) and NeuN (Mouse, MAB377, Millipore, 1:500) diluted in blocking buffer. Following primary antibody incubation, brain sections were washed three times for 10 min in PBS and incubated with secondary antibody (488-Goat anti-rabbit IgG, A11034, Invitrogen, 1:1000; 555-Goat anti-mouse IgG, A21424, Invitrogen, 1:1000), for 2h at RT. Next, brain sections were washed three times for 10 min with PBS, stained for DAPI (Sigma–Aldrich) for 3 min at RT, and mounted on Superfrost slides (Thermo Scientific) using Shandon™ Immu-Mount™ mounting medium (Thermo Scientific #9990402). Image acquisition was carried out using Olympus confocal microscope (FV1000, Olympus) and performed with the experimenter blinded to the experimental groups. Images were acquired with 20x objective and analyzed with ImageJ software to count the number of cells co-expressing FosB/ΔFosB and NeuN. Four striatal slices (AP distance from bregma approximately between +1.2 to +0.6 mm) were used from each mouse.

Corticosterone quantification:
For quantification of corticosterone levels, blood was collected at zenith phase (8.00pm) by cutting the tail tip. All blood collections were done at least 8 hours after the last stress exposure. Clotted blood was centrifuged at 3000 rpm for 10 min and serum was collected and stored at -80ºC until quantification. Corticosterone measurements were performed with ELISA kits (ab108821, Abcam), according to the manufacturer's instructions.

Splash test:
4-weeks old male *SOM-cre* mice were injected with AAV2-hSyn-DIO-hM3D(Gq)-mCherry (Addgene, 44361-AAV2, titer: 1.5 × 10^13 U/mL) into dorsomedial striatum (Bregma: AP +1.0, ML +/-1.5, DV -2.5 mm) and were randomly assigned into control and stress group. After 21 days of stress protocol, all mice were intraperitoneally injected with saline on day 1 and CNO (2 mg/kg, Sigma Aldrich) on day 2. On each day, splash test was performed 1 h after the injection, where mice were placed in a clean homecage, sprayed with 10% sucrose solution on the dorsal coat, and filmed for 10 min at 60 fps. Grooming behavior was manually scored with EthoLog 2.2 (doi.org/10.3758/BF03200814).

Homecage monitoring of total distance:
4-weeks old male *SOM-cre* mice were injected with AAV2-hSyn-DIO-hM3D(Gq)-mCherry (Addgene, 44361-AAV2, titer: 1.5 × 10^13 U/mL) into dorsomedial striatum (Bregma: AP +1.0, ML +/-1.5, DV -2.5 mm) and were randomly assigned into control (non-stressed) and stress group. After 21 days of stress protocol, control and stressed mice were intraperitoneally injected with saline on day 1 and CNO (2 mg/kg, Sigma Aldrich) on day 2. On both days, 30 minutes after the injection, mice were allowed to freely behave in their homecage while being filmed for 30 min at 60 fps. Mice were tracked in real-time using Bonsai, and total distance travelled was calculated offline.

Full description of the mass spectrometry experiments:

1) Sample preparation

Samples were stored at −80˚C until further processing. Upon thawing, 10 µg of each sample from each experimental group were pooled together, creating two pooled samples for library construction. At this point the same amount of a recombinant protein (Green fluorescent Protein and Maltose-binding periplasmic protein (malE-GFP)) was added to the volume corresponding to 40 µg of each sample and to the pools to serve as internal standard. Laemmli buffer was added to each sample in order to have a final concentration of 5% glycerol, 1.7% SDS, 100 mM DTT and bromophenol blue in 50 mM Tris buffer at pH 6.8. All samples were boiled for 5 minutes and acrylamide was added as an alkylating agent.

All samples and pools were then loaded into a precast gel (4–20% Mini-Protean® TGX™ Gel, Bio-Rad), and the SDS-PAGE was partially run for 20 minutes at 110V [PMID: 25418953]. After SDS-PAGE, proteins were stained with Colloidal Coomassie Blue as previously described [PMID: 19702335].

The lanes were sliced into 3 fractions and the gel pieces were destained using a 50 mM ammonium bicarbonate solution with 30% acetonitrile (ACN) followed by a washing step with water (each step was performed in a thermomixer (Eppendorf) at 1050 rpm for 15 min). The gel pieces were dehydrated on Concentrator Plus/Vacufuge® Plus (Eppendorf). To each gel band 75 µL of trypsin (proteomics grade from Roche, 0.01 µg/µL solution in 10 mM ammonium bicarbonate) were added to the dried gel bands and left for 15 min at 4°C to rehydrate. After this period 75 µL of 10 mM ammonium bicarbonate were added and in-gel digestion was performed overnight at room temperature in the dark. After digestion, peptides were extracted to low binding microcentrifuge tubes (LoBind®, Eppendorf) by sequential addition of three solutions of increasing percentage of ACN (30%, 50%, and 98%) in 1% formic acid (FA). After the addition of each solution, the gel pieces were shaken in a thermomixer (Eppendorf) at 1250 rpm for 15 min. The peptide mixtures were dried by rotary evaporation under vacuum (Concentrator Plus/Vacufuge® Plus, Eppendorf). All the peptides from each sample were pooled together for SWATH analysis; the peptides from the pooled samples were kept separately in the three fractions of the digestion procedure for identification purposes.

The eluted peptides were evaporated and solubilized in mobile phase, aided by ultrasonication using a cup horn device (Vibra-cell 750 watt, Sonics) at 40% amplitude for 2 minutes. Samples were then centrifuged for 5 minutes at 14,100×g (minispin plus, Eppendorf) and analysed by LC-MS/MS.

2) Liquid chromatography coupled to tandem mass spectrometry (LC-MS/MS)

The Triple TOF™ 5600 System (Sciex) was operated in two phases: information-dependent acquisition (IDA); followed by SWATH (Sequential Windowed data independent Acquisition of the Total High-resolution Mass Spectra). Peptides were resolved by liquid chromatography (NanoLC™ 425 System, Eksigent®) on a Triart C18 Capillary Column 1/32" (12 nm, S-3µm, 150 x 0.3 mm, YMC) and using a Triart C18 Capillary Guard Column (0.5 × 5 mm, 3 µm, 12nm, YMC) at 50°C with a 50 min linear gradient from 5 % to 30% of acetonitrile in 0.1 % FA and 5% DMSO at 5µL/min. Peptides were eluted into the mass spectrometer using an electrospray ionization source (DuoSpray™ Source, Sciex®) with a 25 µm internal diameter (ID) hybrid PEEKsil/stainless steel emitter (Sciex®).

Information dependent acquisition (IDA) experiments were performed to each fraction of the pooled samples and the mass spectrometer was set for IDA scanning full spectra (350-2250 m/z) for 250 ms, followed by up to 50 MS/MS scans (100–1500 m/z for 60 ms each – in order to maintain a cycle time of 3.3 s). Candidate ions with a charge state between +1 and +5 and counts above a minimum threshold of 100 cps were isolated for fragmentation and one MS/MS spectra was collected before adding those ions to the exclusion list for 15 seconds (mass spectrometer

operated by Analyst® TF 1.8.1, Sciex). Rolling collision energy was used with a collision energy spread of 5.

The SWATH setup was essentially as in Gillet et al [PMID: 22261725], with the same chromatographic conditions used for SWATH and IDA acquisitions. For SWATH-MS based experiments, the mass spectrometer was operated in a looped product ion mode. The SWATH-MS setup was designed specifically for the samples to be analyzed (Sup.Table 1 of the present document), in order to adapt the SWATH windows to the complexity of this batch of samples. A set of 168 windows of variable width (containing 1 m/z for window overlap) was constructed covering the precursor mass range of 350-2250 m/z. A 50 ms survey scan (350-1250 m/z) was acquired at the beginning of each cycle for instrument calibration and SWATH MS/MS spectra were collected from 100–1500 m/z for 19 ms resulting in a cycle time of 3.3 s from the precursors ranging from 350 to 1250 m/z. The collision energy for each window was determined according to the calculation for a charge +2 ion centered upon the window with variable collision energy spread (CES) according with the window.

3) Protein identification and relative quantification:

Specific library of precursor masses and fragment ions were created by combining all files from the IDA experiments and used for subsequent SWATH processing. The library was obtained using ProteinPilot™ software (v5.0.2.0, Sciex), with the following search parameters: reviewed entries for *mus musculus* from SwissProt database (downloaded in January 2021) to which the sequence of malE-GFP was added; acrylamide alkylated cysteines as fixed modification; and the gel based special focus option. An independent False Discovery Rate (FDR) analysis using the target-decoy approach provided with ProteinPilot™ software was used to assess the quality of the identifications, and positive identifications were considered when identified proteins and peptides reached a 5% local FDR. [PMID: 19523214, PMID: 18700793]

Data processing was performed using SWATH™ processing plug-in for PeakView™ (v2.2, Sciex), briefly peptides were selected from the library using the following criteria: (i) the unique peptides for a specific targeted protein were ranked by the intensity of the precursor ion from the IDA analysis as estimated by the ProteinPilot™ software, and (ii) Peptides that contained biological modifications and/or were shared between different protein entries/isoforms were excluded from selection. Up to 15 peptides were chosen per protein, and SWATH quantitation was attempted for all proteins in the library file that were identified below 5% local FDR from ProteinPilot™ search. Peptide's retention time was adjusted by using the malE-GFP peptides. In SWATH™ Acquisition data, peptides are confirmed by finding and scoring peak groups, which are a set of fragment ions for the peptide. Up to 5 target fragment ions were automatically selected and the peak groups were scored following the criteria described in Lambert et al [PMID: 24162924]. Peak group confidence threshold was determined based on an FDR analysis using the target-decoy approach and 1% extraction FDR threshold was used for all the analyses. Peptides that met the 1% FDR threshold in at least three replicates of one experimental group were retained, and the peak areas of the target fragment ions of those peptides were extracted across the experiments using an extracted-ion chromatogram (XIC) window of 5 minutes. Protein levels were estimated by summing all the transitions from all the peptides for a given protein [PMID: 24162925] and normalized to the total intensity of the sample at the protein level.

The mass spectrometry proteomics data have been deposited to the ProteomeXchange Consortium via the PRIDE [PubMed ID: 34723319] partner repository with the dataset identifier PXD031193.

Sup. Table 1 – Information about SWATH windows.

Window	Mass Interval (Da)	Window Width (Da)	CES		Window	Mass Interval (Da)	Window Width (Da)	CES
**1**	349.5 - 394.1	44.6	5		**51**	579.7 - 583.7	4	5
**2**	393.1 - 415.3	22.2	5		**52**	582.7 - 586.7	4	5
**3**	414.3 - 427	12.7	5		**53**	585.7 - 589.7	4	5
**4**	426 - 431.9	5.9	5		**54**	588.7 - 592.7	4	5
**5**	430.9 - 436	5.1	5		**55**	591.7 - 595.7	4	5
**6**	435 - 439.6	4.6	5		**56**	594.7 - 598.7	4	5
**7**	438.6 - 443.2	4.6	5		**57**	597.7 - 601.7	4	5
**8**	442.2 - 446.3	4.1	5		**58**	600.7 - 604.7	4	5
**9**	445.3 - 449.9	4.6	5		**59**	603.7 - 607.7	4	5
**10**	448.9 - 453.1	4.2	5		**60**	606.7 - 610.7	4	5
**11**	452.1 - 456.2	4.1	5		**61**	609.7 - 613.7	4	5
**12**	455.2 - 459.4	4.2	5		**62**	612.7 - 616.7	4	5
**13**	458.4 - 462.4	4	5		**63**	615.7 - 619.7	4	5
**14**	461.1 - 465.2	4.1	5		**64**	618.7 - 622.7	4	5
**15**	464.2 - 468.4	4.2	5		**65**	620.9 - 624.9	4	5
**16**	467.4 - 471.4	4	5		**66**	623.1 - 627.1	4	5
**17**	470.1 - 474.2	4.1	5		**67**	625.8 - 629.8	4	5
**18**	473.2 - 477.2	4	5		**68**	628.1 - 632.1	4	5
**19**	475.9 - 480.1	4.2	5		**69**	630.8 - 634.8	4	5
**20**	479.1 - 483.1	4	5		**70**	633 - 637	4	5
**21**	481.8 - 485.8	4	5		**71**	635.7 - 639.7	4	5
**22**	484.5 - 488.6	4.1	5		**72**	638.4 - 642.4	4	5
**23**	487.6 - 491.6	4	5		**73**	641.1 - 645.1	4	5
**24**	490.3 - 494.9	4.6	5		**74**	643.8 - 648	4.2	5
**25**	493.9 - 499	5.1	5		**75**	647 - 651	4	5
**26**	498 - 503.5	5.5	5		**76**	649.7 - 653.7	4	5
**27**	502.5 - 507.5	5	5		**77**	652.4 - 656.5	4.1	5
**28**	506.5 - 512	5.5	5		**78**	655.5 - 659.7	4.2	5
**29**	511 - 516.1	5.1	5		**79**	658.7 - 663.3	4.6	5
**30**	515.1 - 520.1	5	5		**80**	662.3 - 666.9	4.6	5
**31**	519.1 - 523.7	4.6	5		**81**	665.9 - 670.5	4.6	5
**32**	522.7 - 527.8	5.1	5		**82**	669.5 - 674.1	4.6	5
**33**	526.8 - 530.9	4.1	5		**83**	673.1 - 677.7	4.6	5
**34**	529.9 - 534.1	4.2	5		**84**	676.7 - 681.3	4.6	5
**35**	533.1 - 537.1	4	5		**85**	680.3 - 684.9	4.6	5
**36**	535.8 - 539.8	4	5		**86**	683.9 - 688.5	4.6	5
**37**	538.5 - 542.5	4	5		**87**	687.5 - 692.1	4.6	5
**38**	540.7 - 544.7	4	5		**88**	691.1 - 696.1	5	5
**39**	543.7 - 547.7	4	5		**89**	695.1 - 700.6	5.5	5
**40**	546.7 - 550.7	4	5		**90**	699.6 - 704.7	5.1	5
**41**	549.7 - 553.7	4	5		**91**	703.7 - 708.7	5	5
**42**	552.7 - 556.7	4	5		**92**	707.7 - 712.3	4.6	5
**43**	555.7 - 559.7	4	5		**93**	711.3 - 715.5	4.2	5
**44**	558.7 - 562.7	4	5		**94**	714.5 - 719.1	4.6	5
**45**	561.7 - 565.7	4	5		**95**	718.1 - 722.7	4.6	5
**46**	564.7 - 568.7	4	5		**96**	721.7 - 725.8	4.1	5
**47**	567.7 - 571.7	4	5		**97**	724.8 - 729.4	4.6	5
**48**	570.7 - 574.7	4	5		**98**	728.4 - 733	4.6	5
**49**	573.7 - 577.7	4	5		**99**	732 - 736.2	4.2	5
**50**	576.7 - 580.7	4	5		**100**	735.2 - 739.2	4	5

Window	Mass Interval (Da)	Window Width (Da)	CES
**101**	737.9 - 742	4.1	5
**102**	741 - 745	4	5
**103**	743.7 - 747.9	4.2	5
**104**	746.9 - 751	4.1	5
**105**	750 - 754	4	5
**106**	752.7 - 756.9	4.2	5
**107**	755.9 - 760	4.1	5
**108**	759 - 763.2	4.2	5
**109**	762.2 - 766.2	4	5
**110**	764.9 - 769	4.1	5
**111**	768 - 772.6	4.6	5
**112**	771.6 - 775.8	4.2	5
**113**	774.8 - 779.4	4.6	5
**114**	778.4 - 783	4.6	5
**115**	782 - 786.1	4.1	5
**116**	785.1 - 789.3	4.2	5
**117**	788.3 - 792.4	4.1	5
**118**	791.4 - 795.6	4.2	5
**119**	794.6 - 799.2	4.6	5
**120**	798.2 - 802.8	4.6	8
**121**	801.8 - 807.3	5.5	8
**122**	806.3 - 811.3	5	8
**123**	810.3 - 815.8	5.5	8
**124**	814.8 - 820.3	5.5	8
**125**	819.3 - 824.8	5.5	8
**126**	823.8 - 829.3	5.5	8
**127**	828.3 - 833.8	5.5	8
**128**	832.8 - 838.3	5.5	8
**129**	837.3 - 843.3	6	8
**130**	842.3 - 848.2	5.9	8
**131**	847.2 - 853.2	6	8
**132**	852.2 - 857.7	5.5	8
**133**	856.7 - 861.7	5	8
**134**	860.7 - 866.2	5.5	8
**135**	865.2 - 870.7	5.5	8
**136**	869.7 - 875.2	5.5	8
**137**	874.2 - 880.2	6	8
**138**	879.2 - 884.7	5.5	8
**139**	883.7 - 889.2	5.5	8
**140**	888.2 - 894.1	5.9	8
**141**	893.1 - 898.6	5.5	8
**142**	897.6 - 903.1	5.5	8
**143**	902.1 - 908.1	6	8
**144**	907.1 - 913	5.9	8
**145**	912 - 919.3	7.3	8
**146**	918.3 - 927.9	9.6	8
**147**	926.9 - 936.4	9.5	8
**148**	935.4 - 945.4	10	8
**149**	944.4 - 955.3	10.9	8
**150**	954.3 - 965.2	10.9	8

Window	Mass Interval (Da)	Window Width (Da)	CES
**151**	964.2 - 975.6	11.4	8
**152**	974.6 - 986.8	12.2	8
**153**	985.8 - 999.4	13.6	8
**154**	998.4 - 1011.6	13.2	10
**155**	1010.6 - 1023.3	12.7	10
**156**	1022.3 - 1036.8	14.5	10
**157**	1035.8 - 1051.6	15.8	10
**158**	1050.6 - 1067.4	16.8	10
**159**	1066.4 - 1084.5	18.1	10
**160**	1083.5 - 1103.4	19.9	10
**161**	1102.4 - 1121.4	19	10
**162**	1120.4 - 1139.8	19.4	10
**163**	1138.8 - 1159.6	20.8	10
**164**	1158.6 - 1181.7	23.1	10
**165**	1180.7 - 1205.1	24.4	10
**166**	1204.1 - 1228	23.9	10
**167**	1227 - 1249.6	22.6	10
**168**	1248.6 - 1252.6	4	10
